# An integrative approach for the analysis of risk and health across the life course: challenges, innovations, and opportunities for life course research

**DOI:** 10.1007/s44155-023-00044-2

**Published:** 2023-07-17

**Authors:** Sascha Zuber, Laura Bechtiger, Julien Stéphane Bodelet, Marta Golin, Jens Heumann, Jung Hyun Kim, Matthias Klee, Jure Mur, Jennie Noll, Stacey Voll, Patrick O’Keefe, Annekatrin Steinhoff, Ulf Zölitz, Graciela Muniz-Terrera, Lilly Shanahan, Michael J. Shanahan, Scott M. Hofer

**Affiliations:** 1 Institute On Aging & Lifelong Health, University of Victoria, Victoria, BC Canada; 2 Center for the Interdisciplinary Study of Gerontology and Vulnerability, University of Geneva, Geneva, Switzerland; 3 Jacobs Center for Productive Youth Development, University of Zürich, Zürich, Switzerland; 4 University of Luxembourg, Esch-sur-Alzette, Luxembourg; 5 University of Edinburgh, Edinburgh, Scotland; 6 Pennsylvania State University, State College, PA USA; 7 Department of Neurology, Oregon Health & Science University, Portland, OR USA; 8 University Hospital of Child and Adolescent Psychiatry and Psychotherapy, University of Bern, Bern, Switzerland; 9 Ohio University, Athens, OH USA; 10 Department of Psychology, University of Zürich, Zürich, Switzerland; 11 Department of Sociology, University of Zürich, Zürich, Switzerland

**Keywords:** Risk factors, Health, Life course, Epidemiology, Integrative data analysis, Data harmonization, Data synthesis

## Abstract

Life course epidemiology seeks to understand the intricate relationships between risk factors and health outcomes across different stages of life to inform prevention and intervention strategies to optimize health throughout the lifespan. However, extant evidence has predominantly been based on separate analyses of data from individual birth cohorts or panel studies, which may not be sufficient to unravel the complex interplay of risk and health across different contexts. We highlight the importance of a multi-study perspective that enables researchers to: (a) Compare and contrast findings from different contexts and populations, which can help identify generalizable patterns and context-specific factors; (b) Examine the robustness of associations and the potential for effect modification by factors such as age, sex, and socioeconomic status; and (c) Improve statistical power and precision by pooling data from multiple studies, thereby allowing for the investigation of rare exposures and outcomes. This integrative framework combines the advantages of multi-study data with a life course perspective to guide research in understanding life course risk and resilience on adult health outcomes by: (a) Encouraging the use of harmonized measures across studies to facilitate comparisons and synthesis of findings; (b) Promoting the adoption of advanced analytical techniques that can accommodate the complexities of multi-study, longitudinal data; and (c) Fostering collaboration between researchers, data repositories, and funding agencies to support the integration of longitudinal data from diverse sources. An integrative approach can help inform the development of individualized risk scores and personalized interventions to promote health and well-being at various life stages.

## Introduction

A variety of fields are concerned with how exposure to risk factors affects health and chronic disease across the life course, often with the recognition that exposures and their consequences occur over many decades of life [1]. This insight has also been key to investigations in life course epidemiology (LCE) [2, 3], which recognizes various models of how repeatedly measured risks are associated with outcomes of health and disease. Although the most prominent models of LCE share certain theoretical communalities, they mainly differ regarding the central role of *when* and *how often* risk factors occur and are measured. For example, *accumulation models* suggest that all measurement occasions are important and that exposure to risks starts early in life, accumulating across the life course [4, 5]. In contrast, *sensitive period models* postulate that more than one, but not all, measurement occasions are important, and they stress the importance of the timing of exposure to certain risks for the subsequent development [1, 6]. Relatedly, *critical period models* focus on the importance of one measurement occasion in specific phases of individuals’ life course trajectories [7, 8]. Finally, *recency models* posit that the most recent measurement may be most strongly associated with the outcome [9]. Importantly, these models can also incorporate mediators (i.e., chains or “cascades” of risk) or interactive patterns among repeated risks (called social mobility models) to model associations of risk exposures at various points of the life course with outcomes [10, 11].

Across the different LCE models, well-studied risk factors include indicators and composites of socioeconomic position, social integration, employment status and psychosocial working environment, health behaviors, body mass index, health systems, and stressors including early-life adversity. In turn, these risk factors predict symptoms and disease states, behavioral indicators, and psychological states indicative of distress, biomarkers of health, cognitive performance, physical limitations, physiological systems (e.g., metabolic functioning at various stages of the life course), and ultimately, mortality. Although empirical studies testing LCE models have provided tremendous insights on how exposure to risk affects health-related outcomes across the life course, they also present certain limitations regarding the generalizability of their findings. Most importantly, this type of research is typically restricted to a) separate cohort or panel studies from a single or relatively few countries, and b) a limited age period or range of age.

In the present study, we lay out limitations that are often inherent to LCE when conducted in single-study contexts. We then highlight the need for and potential of moving towards multi-study analyses in LCE, and we illustrate potential challenges that can arise when conducting research in cross-study settings. Next, as core focus of this theoretical review, we present a series of methodological advancements that can address many of these concerns. In more detail, we will discuss new, innovative approaches to data storing, data sharing, data integration and synthesis, and data analyses in multi-study cross-country contexts. Ultimately, one central aim of moving from single-study research to building a multi-study evidence base is to improve health, for example, by applying better-informed individual risk scores and personalized interventions. Because of their rigor, potential for developmental breadth and measurement depth, and ease of interpretation, LCE models hold considerable promise for increasing the use of science in policy making. This is especially the case if, through innovation, LCE models can become increasingly accessible to researchers. Hence, we conclude our review by suggesting an integrative approach based on international, age-period-cohort data that combines developmental breadth and measurement depth on multiple risk factors and various outcomes.

### Limitations of single-study LCE research

One of the major limitations of current LCE approaches is that models of risk and health are predominantly tested with single-study data in an isolated context rather than across multiple datasets. Typically, each study examines the hypothesized developmental pathways with data stemming from a singular type of study design, that is, either from a birth cohort or from other longitudinal designs such as panel studies. Studies of *birth cohorts* (BC) focus on cohorts of individuals who were born in a specific time-period and follow individual developmental trajectories through repeated measurements, typically spread across many decades, which has the advantage of covering a large developmental breadth. *Panel studies* (PS) typically follow one or multiple groups of individuals (e.g., of different ages or born in different periods) across shorter periods of time and therefore often have the advantage of being able to cover a wider range of measurements per assessment time (i.e., they have more measurement depth).

**Fig. 1:**
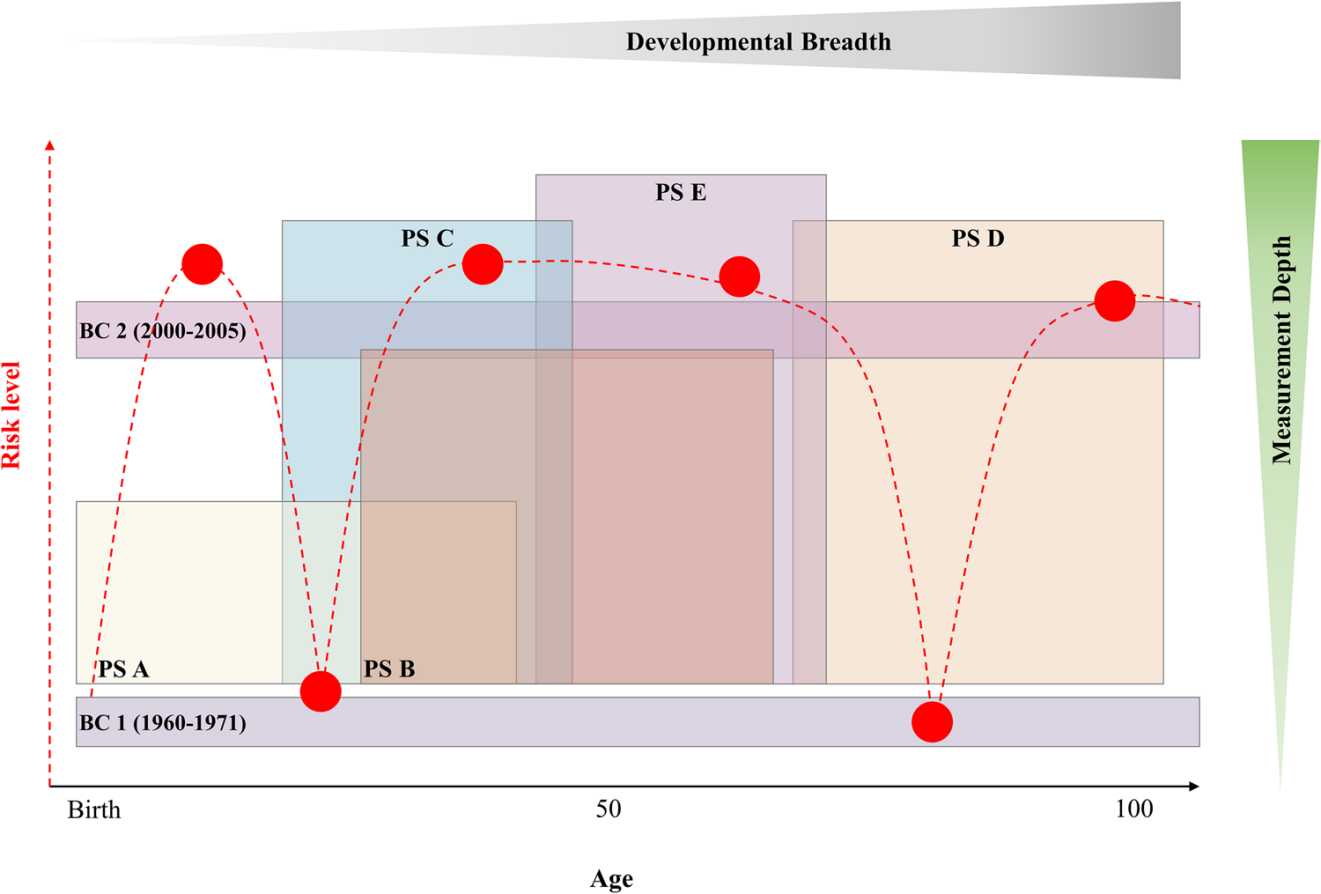
Comparison of birth cohort and panel studies in terms of developmental breadth, measurement depth, and captured risk levels across the life course. *Note.* BC = birth cohorts; PS = longitudinal studies; Numbers 1 and 2 indicate example of different birth cohorts; letters A to E indicate examples of different panel studies. The dotted red line denotes a pattern of potential changes in risk across different ages congruent with life course epidemiological models (e.g., critical or sensitive period) red dots illustrate potential measurement occasions that may (versus may not) be captured by the specific studies

Both BC and PS have provided crucial insights on risk factors and health across the life course, but their data also presents certain limitations, namely that BC usually present limited measurement depth and that PS present a more limited developmental breadth. For illustration, Fig. 1 compares BC and PS in terms of developmental breadth, measurement depth, and risk levels captured across the life course. The Figure also highlights how BC and PS could be combined to increase the breadth and depth of life course risk models. BC 1 and 2 are examples of population-based epidemiological studies with impressive representational breadth of the study sample’s development across a broad age range, but with limited measurement depth in terms of the number and types of variables that were assessed. PS A to E are prospective, longitudinal studies with limited breadth in terms of developmental representation, but with larger sets of variables at varying levels of measurement depth. PS A, for example, covers birth through early-midlife perhaps with only psychosocial depth. PS B covers adolescence through midlife but perhaps adds biological depth in terms of genomics. PS C and D cover differing developmental stages, but have similar levels of measurement depth, for example, including even greater biological depth such as stress and inflammatory biomarkers. PS E focuses on mid- to early late-life and is the most comprehensive in terms of measurement depth assessing psychosocial and biological variables, but also adding neighborhood characteristics and intergenerational transmission.

The dotted red line denotes a pattern of potential changes in risk (e.g., critical or sensitive period) whereas the red dots represent specific measurement occasions where risk may (or may not) have been assessed by the different studies. Risk levels do not inherently have to be connected to measurement depth or developmental breadth. Yet, as illustrated by the red dots, certain risk levels might have been captured by multiple studies, whereas others are uniquely detected by one or a few studies. Thus, examining these studies separately may provide only limited, piecewise information regarding the overarching life course trajectory of risk levels and therefore may pose significant challenges, for example, when examining specific questions regarding critical versus sensitive periods [12]. Most importantly, Fig. 1 also highlights that the risk level trajectory across the life course could only be covered comprehensively when combining data across multiple studies and incorporating alternative longitudinal designs (e.g., ecological momentary assessments). For example, only examining data from both BC 1 and 2 may have underestimated the developmental peaks of risk levels from adolescence to old age and suggested a rather stable, flat risk trajectory across most life periods with a steep increase of risk in old age. Similarly, only focusing on data from PS D and E may have missed short-term decreases in risk levels due to particular events or circumstances that may have occurred at that specific life period (e.g., entering into retirement). Importantly, high risk levels in older age (PS D) could have been detected earlier when also considering data from other studies (e.g., PS C). Unfortunately, it is also possible that certain exposures to risk are not covered by any of the available studies, as represented by the red dot in the top left corner of Fig. 1.

In addition to the limited representativeness of risk trajectories across the life course, when examined separately, individual cohort or panel studies often also do not have the statistical power, specific data items, sufficient duration of follow-up or appropriate design needed to fully address cutting-edge research questions on life course risk and resilience. The limited availability of continuous data that has measurement depth and covers long stretches of the life course often hampers our ability to evaluate life course questions of risk, resilience, and protection using data from a single cohort study. Indeed, although citation counts for research focusing on LCE have increased markedly in the past decade [13], there are surprisingly few empirical studies that attempt to systematically adjudicate among LCE’s central models of critical and sensitive periods and accumulation.

So far, three principal strategies have been used to test such models. These tests usually involve the use of linear models and (1) compare a null model with models that include terms representing life course hypotheses [14], or they (2) compare a saturated model that includes terms for all life course hypotheses with simpler models [15], or they (3) compare effects of repeated risks with expected “reference” effect sizes given a specific life course hypothesis [12, 16]. All such strategies assume that conclusions are not sensitive to the ages covered by the measurement occasions or to the cohorts being studied. That is, they assume that the inclusion of additional earlier or later waves of data or birth cohorts, in addition to the observed data, would not alter conclusions. For example, a study based on samples PS A or B (see Fig. 1) would not reach different conclusions had any of the studies PS C to E also been available. This problem is especially vexing when the final measurement occasion of risk predicts the outcome (thereby suggesting recency) because such a model cannot, without additional waves of data, resolve whether it is part of a critical, sensitive period, or accumulation model. Thus, the testing of life course hypotheses ideally includes data that extends beyond the hypothesized ages at which risk is thought to influence health. Indeed, whenever a final measurement occasion is an important predictor of the outcome, additional waves of data are needed to clarify its interpretation.

## Toward integrative analysis of multiple birth cohorts and panel studies

### Challenges in multi-study, integrative LCE research

*Integrative analysis* is a methodological approach that involves combining and analyzing data from multiple sources, with the goal of providing more comprehensive and informative results than would be possible from analyzing each data set separately [17–20]. It typically involves a) defining the research questions and hypotheses to be addressed, b) specifying the variables and data sources to be included in the analysis, and c) selecting appropriate statistical methods to combine and analyze the data. LCE emphasizes the importance of early life exposures and experiences, as well as the cumulative effects of exposures and experiences over time, on health outcomes in later life. Moving towards integrative analysis of multi-study data therefore presents tremendous potential for future research in LCE, because it allows researchers to synthesize data from multiple sources and time points to better understand how risk factors interact across the life course to affect health outcomes. Specifically, integrative analysis can help identify patterns, trends, and relationships that might not be apparent in any one data set, and can lead to more precise and accurate predictions and generalizations.

Coming back to the examples provided in the previous sections illustrates how integrating analyses across multiple BC and PS hold considerable promise for the complex mapping of critical or sensitive periods where opportunities for intervention are at their peak. For example, integrative analyses of PS C to E would provide the most comprehensive accounting of risk – i.e., with the widest developmental breadth and most phenotypic depth from adolescence through late adulthood – and provide opportunities to evaluate historical cohort differences and selectivity. More generally, applying integrative analyses to birth cohort and longitudinal studies combined will advance developments in research on life course risk, resilience, and protective factors by facilitating cohort linkage and data integration. These efforts would maximize breadth and depth of research projects to advance the understanding of the long-term sequelae of early life adverse contexts and lifespan risk, resilience, and protective factors. Integrative analysis can also help address some of the challenges inherent in LCE, such as selection bias, attrition, and missing data. By pooling data from multiple studies, researchers can increase the sample size and improve the statistical power of their analyses, reducing the risk of false positives and false negatives.

To illustrate some of the techniques that will be presented in more detail in the subsequent sections, one specific example of how life course trajectories can be examined in an integrative context with data stemming from independent, largely heterogeneous studies can be found in [21]. To examine the role of early life personality traits on health-related outcomes later in life, the authors used data from two studies that largely differed in terms of how and when predictors were assessed, which specific health outcomes were examined in mid- versus in late-life, and across how many years individuals were followed. Yet, through data harmonization and integrative analyses, these authors were able to show that aligned personality factors in childhood predicted physiological dysfunction in midlife and mortality in late-life. Examining distinguishable risk trajectories in mid- versus in late-life would not have been possible with any of the available single-study designs alone, which highlights the potential of integrative multi-study research to better capture some of the more complex life course research questions about risk.

Furthermore, integrative analysis can facilitate the examination of multiple pathways and mechanisms by which exposures and experiences across the life course impact health outcomes, helping to elucidate the underlying biological, social, and behavioral processes. This can provide important insights for the development of interventions and policies aimed at improving health outcomes across the life course. With the present review, we therefore urge future research to integrate data or results across studies and countries. In the next sections, we highlight challenges and issues that arise when conducting research in multi-study contexts. In separate sections, we then develop on different steps and solutions towards more integrative analyses of multiple BC and PS and we present in-depth discussions on how such analyses could be achieved and optimized for LCE models.

#### Data access and data sharing

An initial hurdle that researchers may face when aiming to conduct multi-study LCE is identifying potentially relevant data within the large pool of existing longitudinal studies. Once the target data is identified, a next hurdle represents obtaining access to data stemming from different studies, research centers, and often also different countries. Indeed, data sharing and cross-country differences in data protection and privacy continue to be a limitation of data access and co-analysis of data. When relevant information is spread across multiple datasets this typically also implies that it is spread across multiple institutions, repositories, and countries, all of which may have their own regulations and policies on how data can be accessed and shared. Thus, even before getting into the challenges of combining multi-study data, a primary challenge may be the obstacles researchers face when accessing or sharing individual-level data. Related to this, a recent study found that although the majority of authors indicate that they are willing to share their data upon request, only 6.8% actually provide their data [22]. This imbalance does not primarily result from researchers’ actual (un)willingness to share data, but more often is the result of the different challenges that arise when requesting to access or share data.

Indeed, researchers face a variety of potential barriers to data sharing in public health, ranging from technical issues to concerns over legality, ethics, or ownership [23]. Consequently, health research stakeholders internationally have emphasized the importance of data cataloging, dissemination, linkage, harmonization and collaborative use of data and samples to advance research [24]. Such efforts would enable timely access to available data and samples, increase potential to share and analyze data across cohorts, and promote a multidisciplinary and collaborative approach to research through multi-study research consortia.

#### Study heterogeneity

After obtaining access to data of multiple cohort or panel studies, one of the fundamental challenges when working in multi-study contexts and aiming to synthesize empirical findings across datasets is the heterogeneity that exists between them [17]. For example, *population heterogeneity* represents demographic differences across the studies, such as countries, culture, health care systems, subgroups of participants (e.g., age groups, sex, ethnicity) and more. Depending on how large differences between studies are, innovative and complex analytical approaches are required to better understand whether results from analyses apply differently among subgroups (e.g., younger vs. older participants) or whether they may generalize across different populations. *Measurement heterogeneity* occurs when different measurement tools are used for assessing outcomes, exposures, and covariates. On the one hand, measurement heterogeneity can strengthen confidence in findings when the same finding is replicated with the use of different measures in multiple studies of smaller sample size. On the other hand, however, measurement heterogeneity can also pose an important challenge for harmonizing measurements across studies [25–27] that would allow investigators to combine different samples into one large sample in order to address specific research questions that cannot be examined with smaller samples. Finally, *analytical heterogeneity* refers to differences across studies in their designs (e.g., how data was collected, which measurement techniques were used), analytic models (e.g., application of different statistical approaches, variable selection techniques, or approaches to handling missing data), sample size, and number of follow-ups. These differences again can pose challenges when the goal is to combine results across multiple studies [28] and can be further examined at the research synthesis phase using meta-regression and other methods to evaluate sources of differences in results [18].

#### Rare risk factors and rare outcomes

In addition to restricted data access and challenges that arise from heterogeneity between studies, another challenge of multi-study research in LCE is that risk prediction models often focus on rare, unfavorable health outcomes (e.g., relatively rare psychiatric disorders). Large-scale longitudinal observational surveys allow investigating health trajectories over the life course with respect to such events. Besides their many strengths, they are accompanied by different issues inherent in longitudinal data collection, such as selective attrition, dropout, or non-random missingness. For risk prediction models, these issues are particularly challenging because they reinforce the rarity of the events of interest. For example, the people most likely to develop a poor health outcome of interest often are also the most likely to drop out of studies earlier compared to healthier individuals. Limited representation of ethnic or other minorities further contributes to the rarity of certain risk factors and/or outcomes, which, as a consequence, reduces generalizability of resulting prediction models and epidemiological findings [29]. Potential risk and protective factors of investigated events consequently become increasingly rare in the sample. Due to these mechanisms, associations of rare risk factors with even rarer outcomes may be obscured or biased towards the null hypothesis of no association. Even in large observational cohort studies, limited representativeness may reinforce rarity with resulting class imbalance further limiting statistical power to detect potential interactions and relationships between risk factors and outcomes [30].

An additional challenge in studying risk factors is that data may even be rarer when focusing on sensitive information. For example, sensitive information such as income, wealth, or medical history, is often not disclosed or only measured through an approximate indicator (e.g., participants indicating their income by choosing between categories of rather wide-ranged salary classes). In turn, those who do not disclose their information systematically differ from those who do [31, 32] and not at random. Consequently, common strategies to treat missing data, such as listwise deletion or multiple imputation, may lead to differential misclassification in prediction models, which needs to be examined with sufficient robustness checks.

Another common challenge in almost all risk factor research are multicollinearity and omitted confounds. Although empirical studies generally focus on a single risk factor, risk factors co-occur and thus cannot, typically, be studied in isolation. This is especially likely for extreme circumstances of risk (e.g., serious physical abuse or sustained material deprivation) which are likely to be strongly associated with later outcomes. Given appreciable correlations among many different types of risk, studying risks separately raises the possibility of omitted confounds. For example, childhood maltreatment is notably associated with parental substance abuse and other psychopathologies, geographic relocations, disorganized neighborhoods, poverty and other socioeconomic stressors, and limited coping strategies and social supports [33]. Even when research strategies can adjust for such confounds or aim to do so by incorporating them into a single risk score, interactive patterns among multiple risks may be obscured and the specific role of a single risk factor may be over- or underestimated. Such multicollinearity tends to impose analytical challenges, for example, by not meeting assumptions of independence that are commonly made in models embedded within the general linear model. In turn, finding appropriate solutions to model collinearity of risk and to identify different risk profiles that differ in their association with the outcome can augment our understanding of how risk factors and outcomes are associated [34]. In this context, clustering of risk factors can also be compared across cultural settings to investigate generalizability [35].

## Innovations in LCE research methods and statistical analysis

**Fig. 2:**
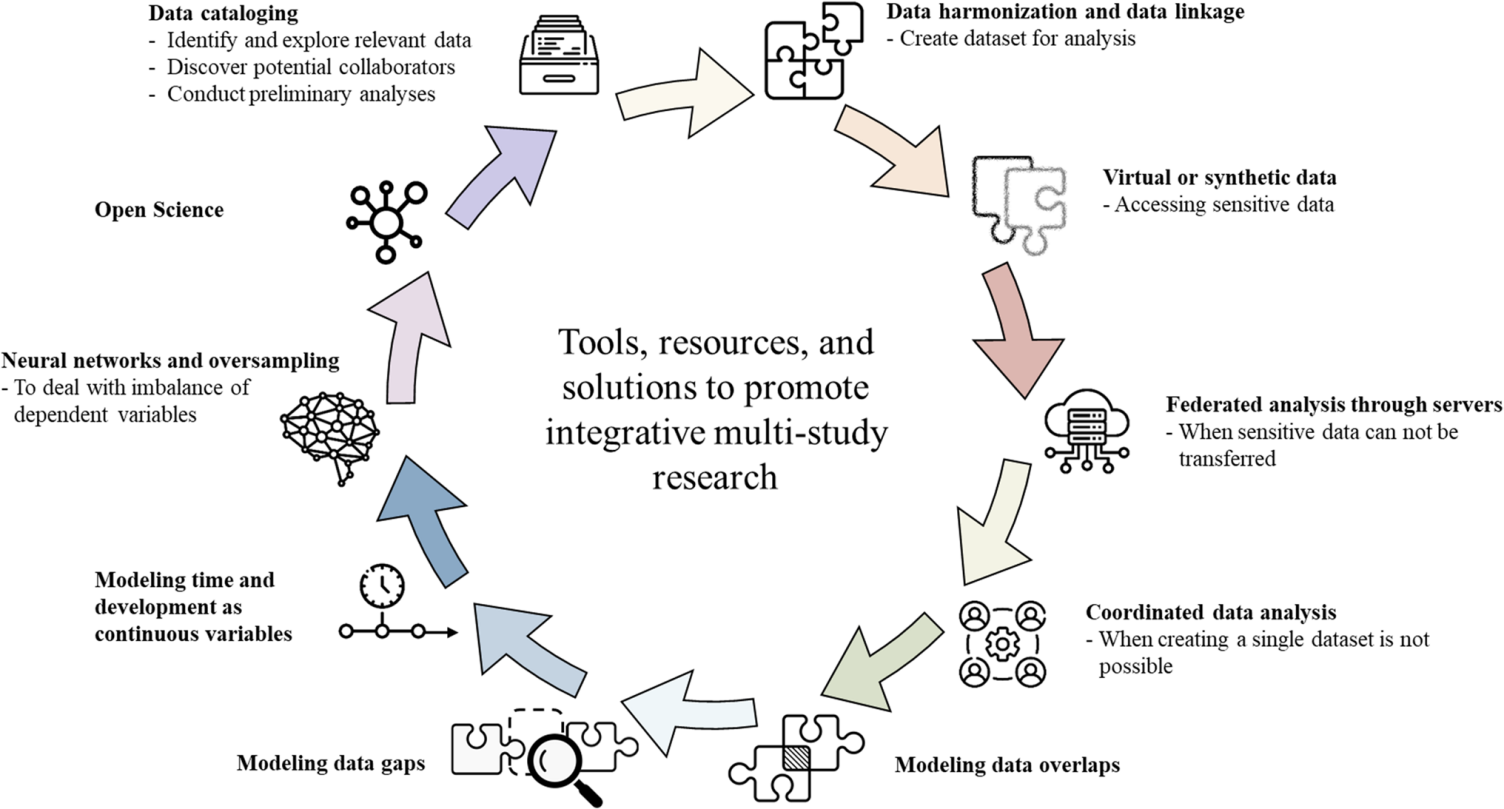
Illustrations of potential workflow to use tools, resources, and solutions to promote integrative multi-study research. *Note.* This figure was created using, editing, or recreating images from Flaticon.com

So far, we have illustrated limitations of single-study research and thereby underlined the importance of conducting LCE in multi-study contexts. We have then highlighted some of the challenges in integrative, cross-study research. In the subsequent sections, we will present a series of innovations in scientific tools, resources, and methodological advancements that can address many of these concerns, such as data cataloging, dissemination, linkage, harmonization, and collaborative use of data. We then present specific modeling techniques that are suited to address challenges that specifically arise when working with longitudinal data in multi-study contexts of LCE: how to model gaps and overlaps in longitudinal data, and how to conceive time and development as continuous rather than discrete variables. Figure 2 represents a possible workflow when conducting multi-study LCE, with different tools, resources, and solutions that—depending on the specific needs, challenges, and research questions of each project—may be applied when aiming to conduct integrative multi-study research. Finally, we will highlight how Open Science practices could feed back into this workflow and thereby ultimately increase available resources and accelerate future LCE research.

### Data cataloging

As illustrated above, an initial challenge that researchers may face when aiming to conduct multi-study research is access to the relevant data. At the very early stages of launching a multi-study project, it may be difficult to identify data that is potentially relevant to address specific research questions, which measurements, time-periods, and populations each individual study covers, how such can be accessed, or which expert teams to contact for specific questions or potential collaborations. One important innovation in this context are *data catalogues*, which gather this type of information on a multitude of studies and make it readily available on online platforms. Typically, such catalogues are organized around a central focus of research (e.g., they include studies from a certain field or studies that target a specific research area) and allow users to discover and search various datasets related to their topic of interest (for an overview, see [36]). Here, for illustrative purposes we will briefly introduce two relevant data catalogues: The Maelstrom Research and The Global Alzheimer’s Association Interactive Network.

*Maelstrom Research* was created as a first, innovative, cross-country and cross-study solution with the aim of cataloging and providing harmonized information across available birth cohorts and other longitudinal studies (see https://www.maelstrom-research.org/) [24]. With the ultimate goal of optimizing the use of study data through collaborative research, Maelstrom Research serves as a central repository of methods, software, and metadata facilitating the emergence and achievement of international research projects co-analyzing data across studies. The Maelstrom Catalogue currently covers over 200 studies, almost 3000 datasets, and includes more than 8 million participants from 55 countries. Maelstrom Research (1) develops methods, promotes best practices, and offers open-source software to support data documentation, harmonization, integration and co-analysis [25], and (2) supports collaborative research making use of harmonized individual participant data. Maelstrom Research engages in collaborations with study networks and consortia to create interactive study catalogues, generate harmonized datasets and develop data infrastructures allowing efficient and secure co-analysis of data across studies.

Along very similar lines, *The Global Alzheimer’s Association Interactive Network* (GAAIN) provides a variety of resources to conduct large-scale research focusing on Alzheimer’s diseases and other dementias (https://www.gaain.org/). With the ultimate goal of improving pre- and interventive health care GAAIN aims to advance disease research by promoting existing resources and facilitating information on and access to different data sets and research groups. GAAIN created an infrastructure to allow faster identification of potential data sources and collaboration partners, and provides resources enabling research to perform comparative data analysis in multi-study contexts. GAAIN includes an interactive online tool through which users can not only explore existing data and create cohorts across multiple data sources but can also conduct preliminary analyses with these data readily available. Through the data discovery platform, researchers can explore clinical, genetic, imaging and other data stemming from various studies and countries. Currently, GAAIN is based on collaborations with over 60 data partners and encompasses over 500,000 subjects and 30,000 measurement attributes.

### Data harmonization and data linkage

As is also illustrated by the data catalogues presented above, in a cross-national context, a key issue is the comparability of outcomes and risk factors based on different measurement instruments that may also differ in language, difficulty, number of items, and range of measurement. Across countries and cultural contexts, studies can vary in how the data was stored (e.g., formatting of datasheet, software), how variables were labelled (e.g., the same construct having different labels as well as similar labels referring to different constructs), or how variables were scaled (e.g., metric versus imperial system; raw vs. normed data), and more. The difficulty in making direct comparisons of the effects of, or on, these measures is that there is no natural metric on which to compare them. This is compounded by between-study heterogeneities, representativeness, and study quality. However, the variety of samples, measurements, contexts, and research designs, particularly in longitudinal aging research, is also an advantage for considering the replicability and generalizability of research findings and permitting an opportunity for broad life course linkage and analyses.

Thus, to examine life course trajectories of risk and health across multiple studies, two key methods are data harmonization and data linkage. *Data harmonization* describes the process of unifying and merging multiple data sources into a single, harmonized dataset [37]. Harmonizing data from cross-country studies enhances the amount of relevant information that is available to researchers and thereby can increase the statistical power, reliability, and generalizability of findings. Exact or psychometric harmonization is usually required for integrative longitudinal data analyses [17, 20, 25, 26].

Whereas data harmonization focuses on combining multiple datasets into one dataset, *data linkage* involves joining data on the same individuals, but from different sources (e.g., linking survey self-report data on smoking with the hospitalization history from administrative data for same individuals). Harmonization may then be used to achieve or improve comparability of the putatively equivalent measures collected by the different studies on different individuals. When either is possible, linkage and harmonization both provide very cost-effective ways to integrate and/or compare data across studies, countries, and birth cohorts. However, because of the complexity and inevitable heterogeneity of the information collected across pre-existing studies and databases, valid comparison or integration of information continues to present major challenges for multi-study LCE [38].

### Virtual or synthetic data and federated analysis

In certain cases challenges to access and obtain data may arise because a potentially relevant study is not captured by any of the available catalogues or because data access is restricted due to country-specific laws or ethical regulations related to sensitive data. While *data anonymization* has been suggested as a solution to some of the problems related to data sharing and data access, there have been reports of successful reidentification attacks [39]. This can be achieved, for example, by searching for specific patterns of relationships across multiple variables that are unique to a particular individual and thereby allow identification.

To face these issues, the use of *virtual* or *synthetic data* represents an alternative approach that bypasses the possibility of reidentifying individuals while maintaining the original pattern of relationships between variables. In this context, different techniques have been developed to statistically replicate the relationships among variables in the original data by creating “digital twins” of the individual participants without identifying the latter. This is usually done with sequential regression models: for each variable, a regression model is developed based on a selection of predictors from preceding variables. This is done sequentially until a model is constructed for each variable [40], in a manner similar to multiple imputation with chained equations [41]. Although such techniques to synthesize data are still relatively novel and may be further developed in the coming years, this is not entirely untrodden ground. Large-scale data-sharing using synthetic data is under way in both the US and the UK, and methods to assess the utility of generated synthetic data have been described [42]. So far, synthetic data has been successfully used in observational studies [43], dementia research [44], and in clinical trials [39, 45].

In addition to sharing virtual or synthetic data, recently developed data platforms allow remote, or federated, analyses of sensitive data through highly secured online servers without actual (physical) sharing of the sensitive data. For example, solutions such as DataSHIELD (Data Aggregation Through Anonymous Summary-statistics from Harmonised Individual levEL Databases) allow researchers to analyze individual level data that could not be shared due to ethical and legal restrictions (https://www.datashield.org/). This is particularly relevant for conducting multi-study research: Once researchers have defined their analysis requests, requests are sent from a central analysis machine to the different sites that hold (parts of) the data. On each site, requested analyses are run in parallel and non-disclosive summary statistics become accessible to the researchers at the central location without the data ever leaving the original host site. With the approach of “taking the analysis to the data, not the data to the analysis”, solutions such as DataSHIELD enable the co-analysis of individual-level multi-study data in contexts where physical data sharing would be difficult because of legal restrictions, duration of application procedure, security issues for the receiving party, or the mere size of the data to be shared [46].

Multi-study synthesis can be particularly challenging when the studies being integrated have significant differences in terms of methodologies used, populations studied, or the contexts within which the studies were conducted. In these cases, differences in results could be attributed to these factors rather than reflecting true differences in the phenomena being studied. There are situations where synthesis might not be advisable:

1. *Inconsistent methodologies* If there are significant differences in the methodologies used across the studies (for example, in terms of data collection methods, measures used, or data analysis techniques), synthesis might not be advisable unless these methodological differences can be carefully accounted for in the analysis.
2. *Different constructs* If the studies are investigating different constructs or phenomena, it may not make sense to synthesize them. Evaluation of harmonization potential is essential before initiating any type of integrative analysis.
3. *Large differences in population or context* If the studies are conducted in significantly different populations or contexts (for instance, one study conducted in a rural low-income population and another in an urban high-income population), synthesis may be inadvisable unless these contextual differences are considered in the analysis.
4. *High heterogeneity* If the variability or heterogeneity across the studies is very high, this could indicate that the studies are not sufficiently similar to be synthesized.

These considerations do not mean synthesis should never be attempted in these situations, but rather that caution should be exercised, and appropriate methods should be used to account for these differences. It is crucial to have a clear understanding of the goals and potential limitations of synthesis before embarking on a multi-study design and synthesis. If the goal is to gain a broad understanding of a phenomenon across diverse contexts and populations, these challenges might be outweighed by the potential benefits. However, if the goal is to obtain precise estimates of a particular effect in a specific context or population, it might be more beneficial to conduct a single, custom-tailored study in that context or population. In the next section, we present coordinated data analysis as additional solution when exact harmonization may be too challenging or not possible.

### Coordinated data analysis

Although harmonizing and pooling data presents many advantages to investigate core questions of LCE, in certain situations unifying datasets may not be possible, for example, because measures, study designs, or certain participant’ characteristics are too disparate to be unified into a single dataset. When exact harmonization and data pooling is not possible different alternative approaches can be used to compare and combine findings across studies which allow examining the same research questions within multiple rather than one dataset. Although multiple approaches can be useful—depending on the specific research question—here we focus on the *coordinated analysis* approach such as proposed by the Integrative Analysis of Longitudinal Studies of Aging (IALSA) [18, 19]. Rather than harmonizing data, coordinated data analysis uses harmonized statistical protocols and analysis plans and thereby has permitted research synthesis based on narrow construct-level measurements.

In detail, the goal of coordinated analysis is to maximize possibilities to replicate and extend empirical findings across studies and time. Instead of pooling data and conducting a single analysis, parallel independent analyses are conducted in a manner that facilitate subsequent comparison and updating of findings. This can be achieved, for example, in research consortia and other collaborative contexts across multiple research groups tackling shared research questions, or by providing open access to research protocols, analysis scripts, and results, so that findings can be revaluated when new, additional data become available. Therefore, coordinated analyses require the initial development of a research protocol that defines how datasets will be prepared (e.g., coding of variables, establishing automatized scripts), analyzed (e.g., determining whether variables should be transformed, standardized, normed, etc.; determining covariates that should be included; determining the most suitable statistical approach), and reported (e.g., transparent and consistent reporting across studies). Coordinated analyses can then be run by the individual research groups or, if possible, in a centralized manner. Results are then reported by independent studies in ways that facilitate cross-study comparison of findings and effect sizes, with comparison of the pattern and magnitudes of effects at the construct level.

Ultimately, key advantages of coordinated analyses are that scientific knowledge accumulates faster, that the stability and generalizability of the findings can be assessed earlier and with more rigor, and—particularly relevant for the study of risk and other infrequent variables—that it increases the statistical power. While the IALSA approach of coordinated analysis has been used primarily for replication and generalizability, this approach can be extended to life course questions, with comparison of results within and across age periods. Although we do not describe those in detail here, alternative or complementary approaches to examine data across studies represent conceptual replications, triangulation of studies, or thematic integration of analyses (for more detailed description of these approaches, see e.g., [47]).

### Modeling data overlaps

Besides challenges with data access and measurement heterogeneities across studies, another challenge that researchers often face when aiming to integrate multiple data sources is that the measurement occasions and life periods that are covered by the different studies either overlap or present gaps. The most common research scenario is represented in Fig. 1, where PS B and PS C largely *overlap* by covering adolescence and part of the adult life, but differ in terms of developmental breadth and measurement depth. In this case, different statistical solutions can be applied, depending on whether the trajectory of the variable of interest is expected or hypothesized to be consistent across the life course—and hence across studies capturing different life periods—or to differ between studies and life periods.

Based on the available evidence and theoretical models of LCE, in many situations it may be possible to defend that the development or risk trajectory of interest is expected to follow a consistent and *specific functional form* across the life course and studies. In this case, it becomes possible to estimate the life course trajectory by constraining the functional form of the model. Specifically, the statistical adaptation then represents a simple interaction model, with an intercept for each study and an interaction effect between each pair of studies. Although this approach seems rather straightforward from a statistical perspective, it is important to highlight that researchers should not rely on a rote application of simple linear models, as part of the goal of life course risk modeling is to assess changes in risk across the life course. As an example of this modeling technique, research from one of our work groups has recently aimed to estimate decline in physical ability across different cohorts [48]. By specifying a particular (nonlinear) functional form, it was possible to provide estimates and predictions of cognitive decline across the later life course although there were significant differences in cognitive ability across cohorts. This illustrates how functional forms can be used as a straightforward approach to examine life course trajectories across cohorts and studies. If researchers are uncertain of the ideal functional form, in certain circumstances model comparison methods may be used to determine the best fitting model. Such a comparison, however, relies on the model being overidentified. This is not a trivial concern, as some models in this domain (e.g., APC models) are routinely just-identified, making model comparisons impossible.

In other contexts, assuming one single specific functional form of development across studies may be inappropriate for the description of change—for example, when studying biological markers of risk and related constructs—because they might lead to unrealistic trajectory shapes across the life course. In this case, *modelling trajectories with splines* can provide more realistic estimates of the life course trajectory. In contrast to assuming a unitary trajectory across the different life periods, this approach allows examining whether the trajectory would be better described by a series of separate functions (i.e., splines) that are joint by knots. To provide an intuitive example: When studying links between sleep and biological markers of stress and health, rather than fitting a single function across the entire life course it is likely that the data could better be fitted by estimating separate functions for different life periods, for example (a) before birth of the first child, (b) after birth of first child, (c) after children enter school age, (d) after children leave parental home, and (e) after retirement.

Because the many heterogeneities between studies represent a significant methodological and conceptual challenge for modelling a single functional form, modelling splines can be particularly useful for multi-study research. In this context, previous work from our group has, for example, applied Bayesian adaptive splines to examine the link between blood pressure and the effect of several risk factors across the entire life course, using data from multiple sources and describing change with cubic and quadratic polynomials [49]. This showed that modelling splines allowed quantifying variability both within and between studies, and examining overall and study specific effects of relevant risk factors. In these and similar situations where a single functional form cannot be assumed, advanced statistical approaches such as spline modelling may provide more adequate estimates of the actual life course trajectory in multi-study contexts.

### Modeling data gaps

**Fig. 3:**
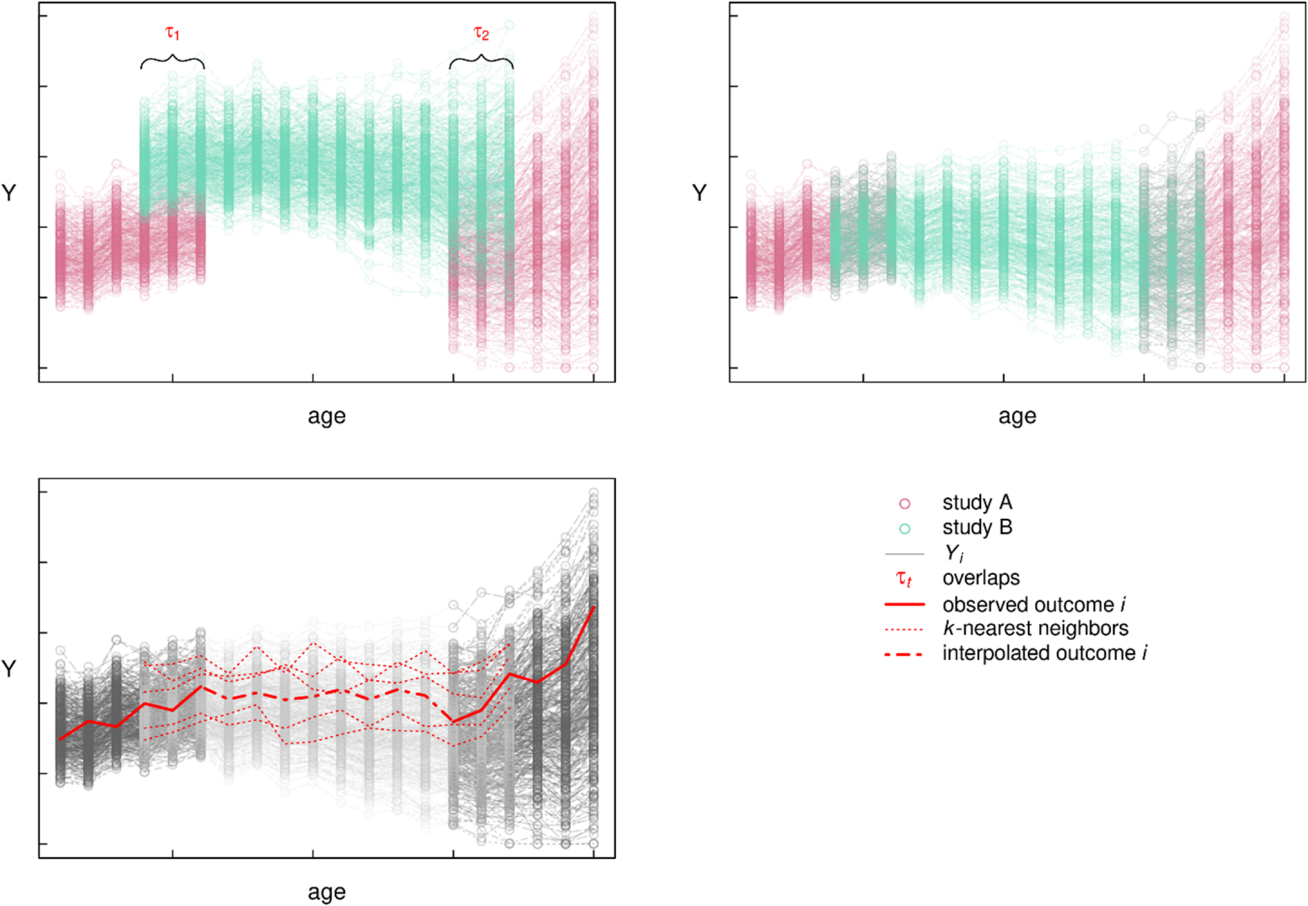
Interpolating parameters for a gap in one study with data from another study using k-nearest neighbors. *Note*. Panel **A**: Study B spans a gap in study A with overlaps *τ*. Panel **B**: Replacing general intercept and slope of study B with those of study A to obtain study B′. Panel **C**: Interpolating individual i in study A using weighted average of *k-*nearest neighbors in overlap with study B′

Another situation that researchers often face when aiming to integrate multiple data sources is that the measurement occasions and life periods that are covered by certain studies present gaps. In these situations, integrative multi-study research presents a unique opportunity to bridge data and thereby allows examining life course developmental questions that could not be answered with a single study. For example, study A in Fig. 3 covers early and late life but presents a significant data *gap* for midlife (panel at top-left). However, from study B, data are available that span the gap of study A, and, importantly, slightly overlap with both sides of the gap. Compared to the functional approaches that we presented for overlapping data, in this case, researchers have more flexibility in their modeling: Unlike having to assume a single or multiple joint functional forms, scenarios with data gaps essentially only require the assumption of a similar functional form of similar individuals across different studies. As the exact functional form does not need to be assumed, this approach leaves more degrees of freedom in the modeling. In fact, encountering data gaps in multi-study contexts is similar to missing data problems in single-study contexts.

One approach to bridging these data gaps is to use the *k-nearest neighbor (KNN) method*, a non-parametric algorithm used for classification and regression tasks that represents straightforward and popular solution across various domains [50, 51]. The algorithm works by finding the *k* closest data points (nearest neighbors) to a new data point and then using the labels of these neighbors to classify or predict the label of the new data point. By using demeaned and detrended data that overlaps between the two studies and by applying a KNN approach, it is possible to integrate data from different studies and to estimate effects that are specific to each study.

In more detail, one could assume that the development of individuals with similar characteristics but from different studies follows a similar functional form. The two studies only differ in terms of intercept and slope but the general functional form of both studies is similar. To integrate the data, the first step is to remove the general intercept and slope from study B (i.e., demeaning and detrending) and replace it with that of study A to obtain study B′ (i.e., aligning the bridging data with the gapping data). Then, to interpolate the gap in the middle ages of individual *i* of study A, one would have to identify the *k* individuals with the most similar trajectories in study B to an individual of study A by calculating Euclidean distances within the overlapping data. The weighted average of the outcomes of those *k* individuals are then the best suitable candidates for convincingly interpolating the outcome of *i* (panel at bottom-left in Fig. 3). Through these steps, KNN approaches can be used for data from longitudinal studies, in which outcomes (e.g., measures of early life adversity, risk, and protective factors) are assessed during different but possibly overlapping age periods and age is a strong predictor. In the next section, we will present an additional approach of how KNN algorithms can be used to solve issues related to time being treated as discrete rather than continuous variable in multi-study longitudinal research.

The influence of historical context and culture is a critical element of life course research and is indeed integral to a multi-study approach. We must add an essential caution in synthesizing results across studies conducted in different historical periods, countries, and cultures. This is a critical dimension of the life course perspective, known as "historical time and place," which refers to the specific socio-historical context in which an individual lives and develops. In the harmonization and interpretation of results, careful attention should be paid to whether a given concept or construct has the same meaning across different cultures or contexts. In addition, the specific historical events and socio-political environments experienced can also significantly shape attitudes, opportunities, risks, and the resources. For example, the accessibility and quality of healthcare, education, and social welfare programs have changed extensively over time and across geographical regions, affecting individuals' ability to mitigate risks. While combining data from independent studies is often justified because of insufficient data otherwise to cover a wide range of age or birth cohort, it is essential that the historical context of these studies and the research synthesis overall is considered. If the respondents from each study did not experience similar historical contexts, these discrepancies could significantly affect the interpretation of the results and result in a biased research synthesis.

### Modeling time and development as continuous variables

Conceptually, LCE models are typically interested in developmental processes that unfold in a continuous manner over time. Yet, empirical studies—particularly large-scale cohort or panel studies—almost always collect data at discrete time occasions. Indeed, in the majority of LCE models discrete measurements are thought to represent potentially complex nonlinear processes that are unobserved. Under certain conditions, these assumptions may be good approximations of the underlying processes. However, in other cases (for instance, when processes are volatile or the number of measurements is scarce) they may lead to biased results and, therefore, misleading conclusions about potential associations of interest. Indeed, in existing formal approaches to model testing, current LCE models with discrete or continuous exposures [14, 16] still treat time as discrete variable and, therefore, diverge from the data generating mechanisms, which represents a limitation of these methods. Modeling time as a continuous variable allows for the examination of the timing and duration of exposures and their effects on health outcomes. This is important because exposures may have different effects depending on the time of occurrence, as well as the duration and frequency of exposure. For example, a person may be exposed to a risk factor for a short period during their life (e.g., maternal substance use during pregnancy; experiencing poverty and unemployment during adolescence or early adulthood), but this exposure may have a lasting effect on their health [52]. Modeling time as a continuous variable enables researchers to capture these dynamic patterns of exposure and their effects on health outcomes. In fact, several methods have been proposed that model discrete, often sparse, measurement occasions as unobserved processes that occur in continuous time (e.g., [53] use a functional regression approach; [54] use B-spline regression).

In this context, *k-nearest neighbors algorithms* (KNN, also see previous section) can also be used to face challenges of discrete time assumptions. Specifically, these algorithms allow assuming that observations are generated by an unobserved latent continuous process, and thus—although risks and outcomes were measured on a limited, select number of occasions and thus represent discrete variables—time and development can be modelled as continuous variables in risk models. The algorithm works by comparing the values of the predictor variables (i.e., time) for a new observation with the values of these variables for the observations in the training data set. The KNN to the new observation are identified, where *k* is a user-defined parameter that specifies the number of nearest neighbors to include in the prediction. The predicted value for the new observation is then calculated based on the values of the outcome variable for the KNN.

By including time as a predictor variable, researchers can gain a better understanding of how the timing and duration of exposures and experiences over the life course can influence health outcomes. KNN algorithms have been successfully applied by different LCE studies, with applications in predicting disease risk and health outcomes based on various early-life exposures and biomarkers. Overall, such work demonstrates the potential utility of KNN algorithms in modeling time as a continuous variable in LCE, providing insights into the associations between various early-life exposures and health outcomes later in life. However, as with any modeling technique, it also highlights that it is important to carefully evaluate the performance of the model and ensure that it is appropriate for the research question at hand.

### Neural networks and oversampling

Besides the potential legal and ethical issues of data sharing/access that we detailed earlier, another significant obstacle to combining datasets represents imbalance of dependent variables. Imbalance of dependent variables describes situations in which the outcome of interest is measured in categories or classes, and there is a significant disparity in the number of observations per class (for example, only 0.1% of the overall sample may be diagnosed with a rare disease of interest, whereas 99.9% of the sample would be identified as “healthy”). This imbalance between response categories imposes challenges for the predictive accuracy of classification models [55].

To overcome this issue, a number of methods have been developed and applied, including both traditional statistical methods as well as newer machine learning methods. For example, if non-responders are sufficiently characterized (e.g., by census data), inverse probability weighting may be employed to account for selection bias due to non-random selection into the sample [56]. Alternatively, in the case where weights are not provided or their calculation is not feasible, training of specialized neural networks with prediction models for underrepresented subsets of participants may be used instead of a one-size fits all prediction approach [57]. However, weighting and other traditional sampling strategies (for example, random oversampling of the minority class, random undersampling of the majority class) often neglect or overemphasize a randomly identified share of the study population. To face these additional challenges, new advancements in machine learning methods may provide more adequate solution to correct imbalance between groups. One key innovation in this area represents the Synthetic Minority Oversampling Technique, which synthesizes new instances of the minority class based on their nearest neighbors (SMOTE) [58, 59]. SMOTE may offer powerful tools to overcome issues related to severe class imbalance when applied carefully and examined thoroughly with respect to calibration and clinical utility [30, 60].

### Open Science in multi-study LCE

Open Science practices in multi-study LCE is an essential step towards improving the quality and impact of research in the field. By promoting transparency, reproducibility, and collaboration, Open Science aligns with the nature of multi-study research, which relies on access to large-scale datasets, technical expertise, and clear reporting. As illustrated in Fig. 2, we strongly believe that Open Science practices allow to feed back into the workflow of integrative multi-study research and thereby enhance the quality, speed, and impact of integrative multi-study research. There are four key reasons why multi-study LCE should adopt Open Science practices:

1. *Enhancing transparency and reproducibility* Open Science allows researchers to share data and analysis code, enabling others to reproduce findings, assess validity, and expand upon previous results. This improves transparency and reproducibility, advancing the field.
2. *Facilitating collaboration* Multi-study LCE often requires collaboration among researchers from various disciplines, institutions, and locations. Open Science enables efficient sharing of data and analysis code, fostering collaboration and promoting a deeper understanding of research questions.
3. *Promoting inclusivity and equity* Open Science reduces barriers to participation, increasing collaboration opportunities across diverse communities. By sharing research data and materials, researchers ensure accessibility to underrepresented groups and improve visibility of datasets, contributing to a diverse and inclusive research community.
4. *Enhancing impact and dissemination* Open Science increases the visibility and accessibility of research, leading to wider dissemination and broader impact on public health policies and practices. This also ensures research is usable by other researchers, resulting in more impactful outcomes.

As the LCE field grows and evolves, it is vital for researchers to adopt and promote Open Science practices to ensure the development of a robust, diverse, and inclusive research community that can generate meaningful and impactful insights. By doing so, the field of LCE can better address the challenges it faces and continue to push the boundaries of scientific knowledge.

## An integrative framework of multi-study LCE: current state of the art and next steps

Risk models are essential tools for prevention and intervention efforts that target many health outcomes across the life course. According to such models, patterns of risk factors early in life increase the likelihood of negative outcomes in later years. Despite the intuitive appeal of risk models, progress in the empirical study of this proposition has been modest across the study of many different outcomes and in different phases of the life course. For example, in suicide research, “predictive ability has not improved across 50 years of research; studies rarely examined the combined effect of multiple risk factors; risk factors have been homogenous over time, with 5 broad categories accounting for nearly 80% of all risk factor tests” ([61], p. 187). The same statement could be made for most fields of risk research in human development, including, for example, the study of psychopathology, physical health, and dementia.

Optimizing research on LCE, early life origins of adult health and the role of lifelong risk, resilience, and protective factors involve maximizing the use of the available cohorts and other longitudinal studies with adequate follow-up and exposure heterogeneity. Integrative data analytic approaches [17–20] are often essential to achieve sufficient statistical power to detect multivariate dynamics, interactions, and subgroup differences and for reliable comparison, cross-validation, or replication across datasets. To answer these requirements, longitudinal study data benefits from being Findable, Accessible, Interoperable and Reusable (FAIR) [62], and more particularly, from being harmonized. Harmonization is required to achieve or improve comparability of the putatively equivalent measures collected by different studies on different individuals. Although the characteristics of the research initiatives generating and using harmonized data vary extensively, the present review has illustrated how all are confronted by similar issues. Having to identify, collate, understand, process, host, and co-analyze data from independent studies is particularly challenging. However, we have seen major advances in data cataloging and access (e.g., IALSA, Maelstrom Research, GAAIN) over the past 10 years and this will be foundational for next steps in life course risk research.

### Synthesis of Models—bringing innovations together to advance LCE

In our review, we have presented innovative approaches that each allow addressing certain – but not all – of the challenges that arise when examining life course risk and health trajectories from a multi-study perspective. Thus, to further increase the potential impact of such multi-study research, these innovations in statistical modeling in LCE and the extension of data bridging for different age periods and countries through various imputations should be used in conjunction to answer the trajectory of risk factors throughout the life course. For instance, with extended data across age periods and relaxing discrete time assumption, one can precisely detect the optimal intervention timing with valid confidence intervals. However, this combination of methods needs to take into account strong correlation between multiple risks and be able to answer how other risk factors unfold over time. Depending on the specific research questions, the theoretical LCE model, and the researchers’ analytical approach, certain variables may be considered primarily as risk factors, whereas—in other contexts—they might also be considered as mediators, modifiers, or as outcomes [63]. Because of the complexity of the temporal dynamics between these risk factors, their integration into models of risk and more generally, LCE approaches, may be challenging and necessitate a large amount of data over time. Triangulation of methods or a kaleidoscopic approach where several models are fitted to the data to examine a question from multiple perspectives may provide insights into some of these complex relationships. Model averaging may be an alternative approach that could inform researchers providing opportunities for integration of results across models [64].

While multi-study model synthesis is indeed a novel and developing area, it is a complex process and it is important to be transparent about the methods used and to thoroughly assess the quality of the models being synthesized (see e.g., [65]). This includes, for instance, examining the assumptions made in each model, the data used, and the fit of the model to the data. Despite these challenges, the synthesis of models offers a promising avenue for understanding complex risk and resilience factors and their trajectories and impacts across the life course.

### Approaches for addressing heterogeneity across studies in an integrative analysis

Identifying common, but also potentially unique, risks across different contexts and populations is a major goal of multi-study designs but also for cumulative science based on independent studies. While reconciling discrepancies between different studies or data sources remains a significant challenge, a multi-study, integrative analysis approach is most useful because it allows isolating differences due to measurement and statistical method and thereby maximizes comparability of independent study results. To discern whether differences are due to different life periods, contexts, or studies, we can use several approaches:

1. *Contextualized analysis* First, it is important to analyze data in their respective contexts. This involves considering factors that may influence the outcomes in each context, such as socio-economic factors, cultural norms, or historical shifts in policy. By identifying these contextual factors, it is possible to better understand the differences in results across studies.
2. *Subgroup analysis*: This approach is based on co-analyzing or contrasting subsets of data based on different characteristics or categories. This could include demographic factors (such as age, gender, race, and more), socio-economic factors, or any other relevant factor. This can help isolate the effects of particular variables and can often elucidate whether differences in results are due to these factors.
3. *Meta-regression* This is a statistical method used to explain the heterogeneity across the results of different studies [66–68]. Meta-regressions could, for example, test whether the magnitude of the observed effects differs based on characteristics of the studies, such as the context in which the study was conducted, the period when the study was conducted, or the methodology used in the study.
4. *Sensitivity analysis* This is a method used to determine how different values of an independent variable can impact a particular dependent variable under a given set of assumptions. Sensitivity analysis can be useful in testing the robustness of the findings across different contexts or populations.
5. *Collaboration and expert consensus* Finally, in multi-study designs, it is often useful to have expert discussions and consensus meetings. These can help to interpret differences across studies by drawing on the collective expertise of researchers.

Although understanding differences across studies in a coordinated or integrative data analysis currently remains a challenging issue, these approaches can be used effectively to advance the analysis of common and unique risk factors. There is certainly still a need for further methodological work to address these issues, and clear guidelines should ideally be established to support researchers in identifying and reconciling systematic differences in results across populations, contexts, and historical time.

### Applying LCE results in clinical and applied settings

Life course epidemiology has the potential to significantly impact clinical and applied developmental settings by informing early identification and intervention strategies, promoting personalized medicine, fostering a holistic approach to patient care, enhancing our understanding of developmental processes, encouraging cross-disciplinary collaboration, and guiding health policy and public health initiatives. LCE can lead to the development of more effective prevention and intervention strategies, targeting at-risk individuals at the earliest possible stages. Indeed, for many fields focusing on the study of health and health care, guidelines for transparent reporting of clinical models exist for prognosis as well as diagnosis [69]. Yet, such guidelines have typically not been developed with a life course perspective, in part, because pediatric and adult speciality fields have limited interactions. However, LCE would benefit from similar considerations about assessment of bias and quality of evidence. For example, a guiding question to develop such norms could be in which context prediction models will be applied, which modeling strategies were employed, and how data characteristics may affect performance across subgroups.

As an example, if a model is used for the prediction of clinical risk, the model should be well-calibrated, accurate, and—in case of a binary prediction such as “diagnosed” versus “diagnosis-free”—it should discriminate consistently and reliably between classes. While many prediction models perform well with respect to accuracy (in terms of sensitivity and specificity), well-established risk scores such as Cardiovascular Risk Factors, Ageing and Dementia (CAIDE) for dementia risk prediction suggest only moderate discrimination [70]. This raises questions about the clinical utility and the ethical consequences of model application for risk prediction in real-world applications. More precisely, even in statistical models that are accurate and discriminate well in training and test data, calibration needs to be considered when these models are applied in clinical contexts. Otherwise, probabilities of predictions may be overestimated, e.g. because of overfitting, leading to misclassification of new observations. Resulting issues regarding the clinical utility of prediction models may, for example, be addressed with an evaluation of the decision curves [71].

Many studies have demonstrated that early life exposures to adverse conditions can have long-term impact on later developmental trajectories and disease risk in adulthood and later life (e.g., [72]; for a comprehensive review see [73]). A challenge, however, is to understand risk pathways, its propagation and dynamics across the life course, and how risk accumulates to impact adult health and aging. The developmental origins of health and disease concept encompasses how, during early life (preconception, pregnancy, infancy, and childhood), the interplay between maternal and environmental factors program fetal and child growth and developmental trajectories, and influence susceptibility to disease in later life, possibly involving the modification in the activity of genes and other forms of biological embedding. However, few single studies have the breadth and depth of data to adequately test life course models of risk.

In order to advance LCE and improve our theoretical understanding of life course mechanisms of risk and health a broader developmental context is required. Evidence needs to be evaluated with respect to underlying characteristics of the data that has been used to train models, the modeling strategies that were employed, and the context of application. Examples from other fields such as medical research may offer powerful solutions and their application in the field of life course risk should be further investigated.

### Models for dynamic scoring of risk

One core goal of LCE is to be able to detect risk and health-related changes early in life, which then allows setting up useful pre- or intervention programs in timely manner and thereby, hopefully, putting individuals on a more favorable developmental trajectory. To support earlier detection of change and risk, repeated measurements are required to establish individual-specific baselines for the reliable detection of within-person dynamics, which can then be stratified into interventions at earlier phases. Largely because of insufficient within-person data, the analysis of within-person dynamics, risk profiles incorporating change parameters, and predictive models for change and change-points in functioning have rarely been uniformly applied to prospective clinical research (but see summary of research on early detection of cognitive impairment; [74]). A better understanding of the dynamics of risk within the health care context are important, as data on within-person change has great potential to improve confidence in diagnosis, direct prevention, and risk reduction efforts, and form the basis for more continuous evaluation of care.

## Conclusion

The present overview illustrates limitations of single-study research in LCE and promotes moving toward integrative multi-study research by presenting specific tools, resources, and solutions to face many of the challenges that arise when working in cross-study and cross-country contexts. As a core principle to the study of risk and health across the life course, LCE recognizes that health outcomes are influenced by multiple factors that interact with each other and that these effects and their interactions may vary across time and age. Analyzing data and combining findings from multiple studies is central to examine these complex life course trajectories and to gain a broader perspective on how risk and health unfold across the life course. By integrating data and results from studies with different designs, outcomes, and populations, multi-study LCE allows examining the robustness and consistency of findings across different populations, countries, and cultures. Similarly, it allows studying the mechanisms by which individual exposures and experiences across the life course impact health outcomes, helping to elucidate the underlying biological, social, and behavioral processes. This can provide important insights for the development and implementation of interventions and policies aimed to improve health outcomes at any age. To summarize, we encourage future research to apply a multi-study integrative analysis approach because it will advance LCE over the next decade in several ways:

1. *Increased sample size and statistical power* Pooling data from multiple studies increases sample size and statistical power and thereby can allow for the detection of smaller effect sizes. Crucial for LCE, this may help identifying risk factors, critical or sensitive periods that would have been missed in single-study analyses. Hence, multi-study LCE can provide more robust and more precise evidence for the associations between factors and health outcomes.
2. *Examination of heterogeneity and generalizability*: Findings from single-study research may be less generalizable to other populations or settings due to various heterogeneities between studies and the studied populations. Multi-study research can help examining the heterogeneity of associations across different populations and settings and assess whether findings hold across different contexts, thereby lead to more generalizable findings. This is central for evidence-based policy making, as it can provide important insights into how the effects of exposures may vary across different life stages and populations.
3. *Identification of common exposures* Combining data from multiple studies—and hence typically from different populations, ages, time periods, countries, cultures, and more – may allow identifying common exposures that are associated with different health outcomes across the life course. This can provide important targets for future research on the biological mechanisms underlying these associations.
4. *Examination of intergenerational effects* Multi-study research can help to examine intergenerational effects, which occur when exposures in one generation affect the health outcomes of subsequent generations. This can provide important insights into the long-term health effects of exposures that occur early in life (see, for example, Consortium on Individual Development; [75]).
5. *Synthesis of evidence* Multi-study research can help to synthesize evidence from multiple sources, including observational studies, randomized controlled trials, and experimental studies. This can provide a more comprehensive understanding of the complex interactions between biological, social, and environmental factors that contribute to health outcomes throughout the life course.
6. *Comparison of theoretical models* Multi-study research allows more accurate comparison of different theoretical models of LCE, because it allows for a more comprehensive understanding of the complex relationships between different factors and health outcomes. In cross-study contexts, researchers can identify commonalities and differences in these interactions and their life course trajectories. This can help to refine and improve theoretical models of life course epidemiology by providing a more accurate estimation of the convoluted interplay between different risk and health variables.
7. *Identification of research gaps* Ultimately, as multi-study research opens new perspectives on risk and health across the life course, it can also help identifying uncharted research gaps in LCE. By synthesizing findings from multiple studies and across diverse datasets, researchers can identify areas where more research is needed and prioritize future research efforts.

Although conducting multi-study research presents many challenges in its own, with the present review we not only highlight the relevance of such undertakings but also propose an in-depth overview of innovative tools, resources, and solutions that meet many of these challenges. We propose an integrative framework that combines the advantages of multi-study data with a life course perspective. This framework can guide research in understanding life course risk and resilience on adult health outcomes by fostering collaboration between researchers, data repositories, and funding agencies to support the integration of longitudinal data from diverse sources.

Typically, each study that is part of an integrative analysis would provide valuable insights on its own, but synthesizing them with other appropriate data could provide a more nuanced and comprehensive understanding of the impact of risk on outcomes across the life course. The challenges in this process might include reconciling different operationalizations of risk and outcomes, accounting for different methods used in data collection and analysis, and interpreting differences in results across different sociopolitical and historical contexts. However, by carefully addressing these challenges with a much richer set of data, it is possible to gain a richer understanding of life course risk that takes into account the complexity of this issue and its manifestation across diverse contexts. While a multi-study approach is undoubtedly time-consuming and challenging, it has the potential to provide a more comprehensive and nuanced understanding of complex issues. However, it is essential to weigh these potential benefits against the resources required and to consider whether a multi-study approach is the most appropriate method given the specific research question and context.

## Data Availability

Not applicable.
